# 3D Printed, Microgroove Pattern-Driven Generation of Oriented Ligamentous Architectures

**DOI:** 10.3390/ijms18091927

**Published:** 2017-09-08

**Authors:** Chan Ho Park, Kyoung-Hwa Kim, Yong-Moo Lee, William V. Giannobile, Yang-Jo Seol

**Affiliations:** 1 Dental Research Institute, School of Dentistry, Seoul National University, 1 Gwanak-ro, Gwanak-gu, Seoul 08826, Korea; 2 Department of Periodontology and Dental Research Institute, School of Dentistry, Seoul National University, 28 Yongon-dong, Chongno-gu, Seoul 110-749, Korea; perilab@snu.ac.kr (K.-H.K.); ymlee@snu.ac.kr (Y.-M.L.); 3 Department of Periodontics and Oral Medicine, School of Dentistry and Department of Biomedical Engineering, College of Engineering, University of Michigan, 1011 North University Ave., Ann Arbor, MI 48109-1078, USA; wgiannob@umich.edu

**Keywords:** 3D printing, ligament, microgroove patterns, biopolymer, tissue engineering

## Abstract

Specific orientations of regenerated ligaments are crucially required for mechanoresponsive properties and various biomechanical adaptations, which are the key interplay to support mineralized tissues. Although various 2D platforms or 3D printing systems can guide cellular activities or aligned organizations, it remains a challenge to develop ligament-guided, 3D architectures with the angular controllability for parallel, oblique or perpendicular orientations of cells required for biomechanical support of organs. Here, we show the use of scaffold design by additive manufacturing for specific topographies or angulated microgroove patterns to control cell orientations such as parallel (0°), oblique (45°) and perpendicular (90°) angulations. These results demonstrate that ligament cells displayed highly predictable and controllable orientations along microgroove patterns on 3D biopolymeric scaffolds. Our findings demonstrate that 3D printed topographical approaches can regulate spatiotemporal cell organizations that offer strong potential for adaptation to complex tissue defects to regenerate ligament-bone complexes.

## 1. Introduction

Fibrous connective tissues in musculoskeletal systems require highly specialized spatiotemporal organizations with physical integration and mineralized tissues for physiological responsiveness under biomechanical stimulations [1]. Specific orientations of fibrous tissues on highly organized collagenous constructs have crucial roles in optimizing various biomechanical and biophysical responses like absorption, transmission or the generation of forces [1,2]. However, diseases or traumatic injuries of the musculoskeletal systems could induce instabilities in multiple tissue interfaces or the loss of their skeletal-supportive functions [1]. In the cases of craniofacial and dental complexes, multiple tissue integrities with coordinated ligaments, the periodontal complex (bone-periodontal ligament (PDL)-cementum), facilitate the optimization of various systematic functional responses to support and position the teeth [3,4,5,6]. In particular, angulated PDLs with spatiotemporal organizations between the teeth and the alveolar bone significantly contribute to masticatory/occlusal stress absorptions and distributions [3,7], as well as the optimization of mineralized tissue remodeling for tooth-periodontium complexes [8]. Therefore, perpendicular/oblique PDL orientations to the tooth-root surfaces add to the functionalization and revitalization of tooth-supportive biofunctional structures.

Various state-of-the-art approaches with micro-/nano-topographical characteristics on 2D substrates have been developed to generate various cell-material interactions [9,10,11] and to regulate cell behaviors, such as cell adhesion, migration, proliferation, differentiation or specific cell organizations [12,13,14]. Beyond 2D perspectives for biomedical applications, additive manufacturing or 3D printing techniques permit spatial designs of specific geometries [15], and many efforts have contributed to manufacturing 3D scaffolding systems for preclinical and clinical scenarios [16,17,18,19]. In our example, fiber-guiding scaffolds particularly promoted periodontal regeneration with tissue compartmentalization and limited PDL organizations to the tooth root surface [20]. In addition to preclinical studies, our first human case study using a patient-specific scaffold manufactured by 3D printing attempted to treat a large periodontal defect and to regenerate periodontal complexes (bone-PDL-cementum) [21]. However, there remain challenging limitations for the spatiotemporally control of perpendicular/oblique angulations of ligamentous bundles for physiological functioning restorations in the ligament-bone complexes.

**Figure 1:**
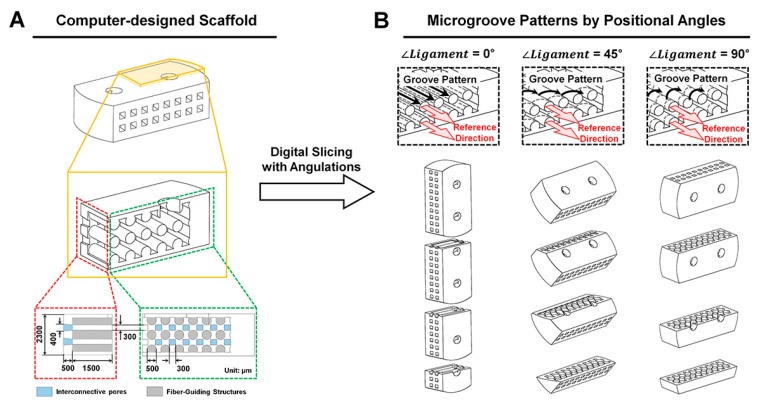
3D designs of engineered ligament scaffolds. (**A**) After designing 3D scaffolds with three-layered ligament-guiding architectures; (**B**) different angles were set for the additive manufacturing. The yellow part was the quarter of the scaffold to show the designed internal architecture. Green and red dash-lined boxes presented the side and the front views of the scaffold with the design parameters.

**Figure 2:**
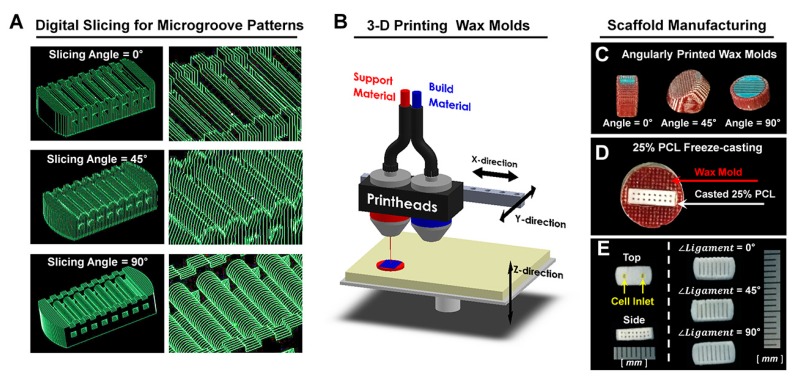
Fabrication of engineered ligament scaffolds. (**A**) The digital slicing procedure created angulated microgroove patterns on ligament architectures; (**B**–**D**) the additive 3D printing system produced dual wax constructs and 25% PCL (poly-ε-caprolactone) was casted after removing blue wax parts; (**E**) PCL scaffolds were shown with similar external architectures.

Herein, we investigated a simple, but precisely controllable method to create 3D printed architectures with cell-responsive, micro-topographies with angulated patterns on spatial scaffolds (Figure 1 and Figure 2). The additive manufacturing system creates micron-scaled, layer-by-layer artifacts, which are generally removed for smooth surface qualities. In this study, the angular-controllable microgrooves (known as artifacts) were actively considered for specific surface patterns, which can be programmed in the digital slicing step of the additive manufacturing procedure (Figure 1B and Figure 2A). The manufacturing strategy for biomimetic microenvironments can enable integrated angulated microgroove patterns on ligament-guiding scaffolds to promote spatiotemporally-directional and anisotropic cellular consolidations.

## 2. Results

**Figure 3:**
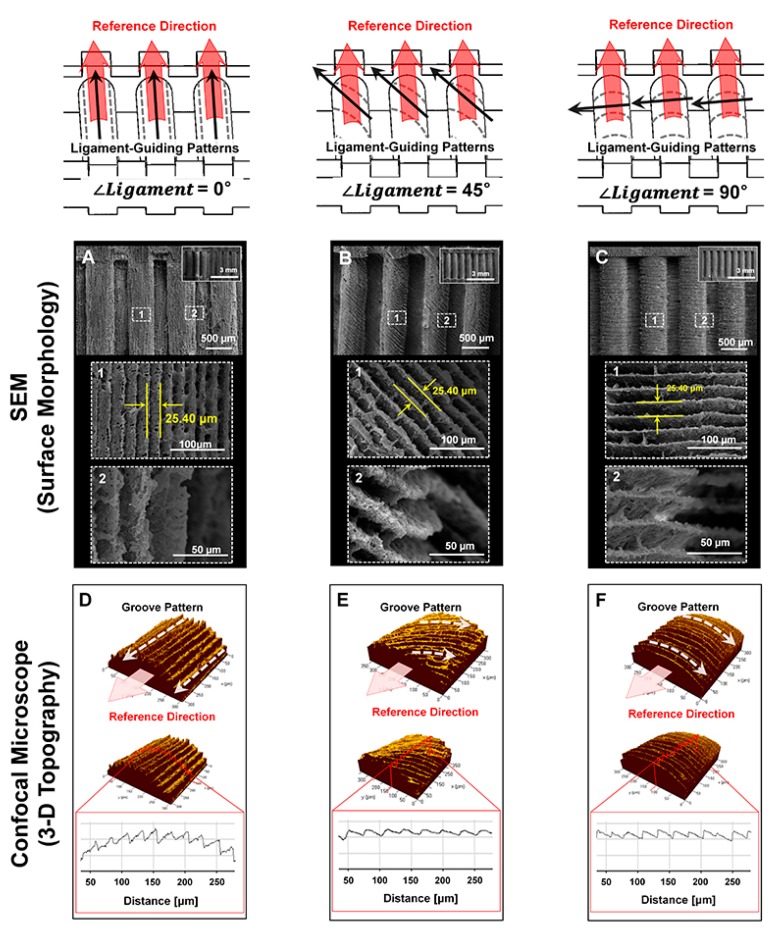
Image analyses of microgroove patterns on ligament architectures of scaffolds. (**A**–**C**) Scanning electron microscope (SEM) qualitatively demonstrated surface morphologies, roughness and angulated microgroove patterns on ligament scaffolds with the distance between microgroove patterns by the digital slicing procedure (25.40 μm); (**D**–**F**) the confocal microscope facilitated topographical investigations for different angulated microgroove patterns to the reference direction. In the red boxes of **D**–**F**, surface topographies and roughness of three different groups (*∠Ligament* = 0°, 45° and 90°) were characterized and profiled using crossed red-lines on the scaffolds. Based on the surface profiles, intervals of microgrooves can be determined with approximately 25.40 μm.

**Figure 4:**
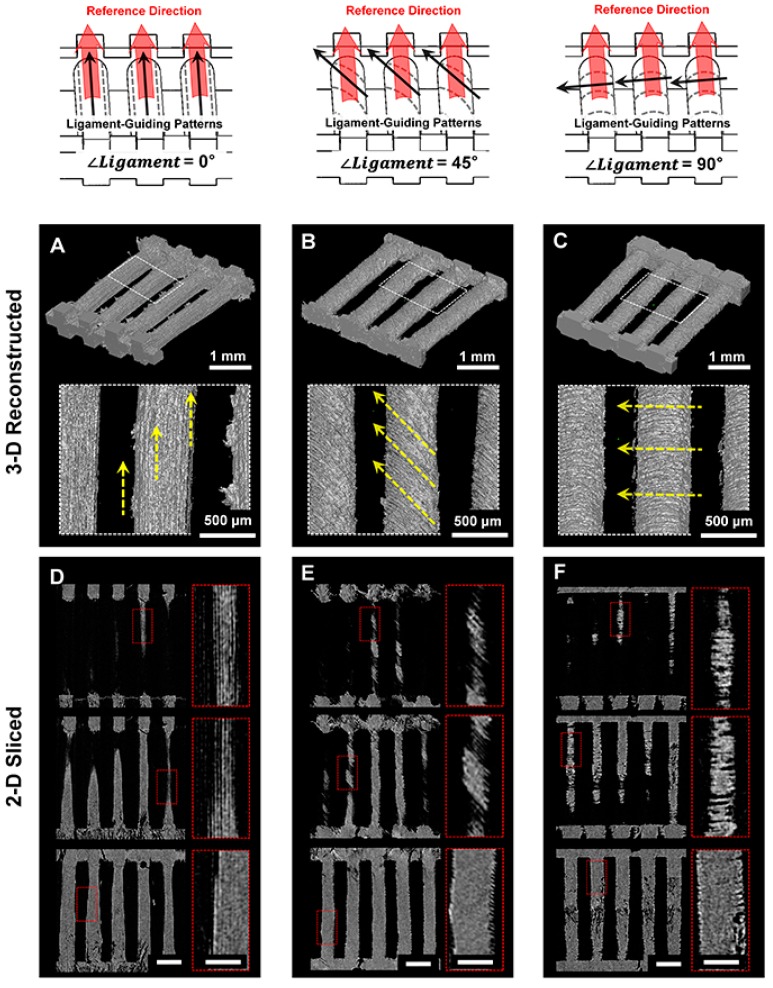
Micro-computed tomographic (micro-CT) images for 3D reconstructed and 2D sliced image analyses. (**A**–**C**) 3D reconstructed spatial patterns were qualitatively analyzed on scaffold surfaces and (**D**–**F**) 2D sliced images demonstrated microgroove directionalities after fabrication of engineered ligament scaffolds. The scale bars in **D**–**F**: 1 mm. The scale bars in the red-dash boxes in **D**–**F**: 250 μm.

Scanning electron microscope (SEM) showed each angulated ligament-guiding, microgroove patterns on the PCL scaffolds at a low magnification and rough surfaces on the microgroove wall-sides at a high magnification (Figure 3A–C). In particular, the surface roughness can be generated by the freeze-casting method with various concentrations of PCL in 1,4-dioxane, and different surface properties of the scaffolds can be significantly characterized for the biological responses of the cells. At this point, due to the difficulty of quantitative measurements of roughness on spatial structures of microscopic topographies, the surface roughness was analyzed using the freeze-casted PCL constructs on flat wax molds (Figure S1). Among the different PCL solutions, the surface roughness by the 20% and 25% PCL solutions showed statistical similarities (Ra (20%) = 2.237 ± 0.727 µm; Ra (25%) = 2.00 ± 0.700 μm; Figure S1), and the 25% PCL solution was significantly considered for the scaffolds to enhance cell attachment and viability [22]. In addition to indirect characterizations of scaffold topographies (Figure S1), noisy section-profiles of microgroove patterns on scaffolds can qualitatively show the surface roughness (Figure 3D–F). Based on geometric assessments, the precise additive manufacturing resulted in high reliability and reproducibility to generate microgroove patterns with angulated slicing procedures. From the micro-CT (micro-computed tomography) results, the microgroove patterns on the referential ligament structures were spatially qualitative in the 3D reconstruction, and the microgroove depths of all of the groups were measured with 2D segmented scaffold images (Figure 4). Based on various structural characterizations of the 3D ligament scaffolds (*∠Ligament* = 0°, 45° and 90°), the additive manufacturing system created highly reproducible and adjustable microgroove structures by the angular slicing procedures. Furthermore, the freeze-casting method of the biopolymeric solution (25% PCL solution in 1,4-dioxane) facilitated roughness to enhance cellular attachments to the biomaterial surface.

**Figure 5:**
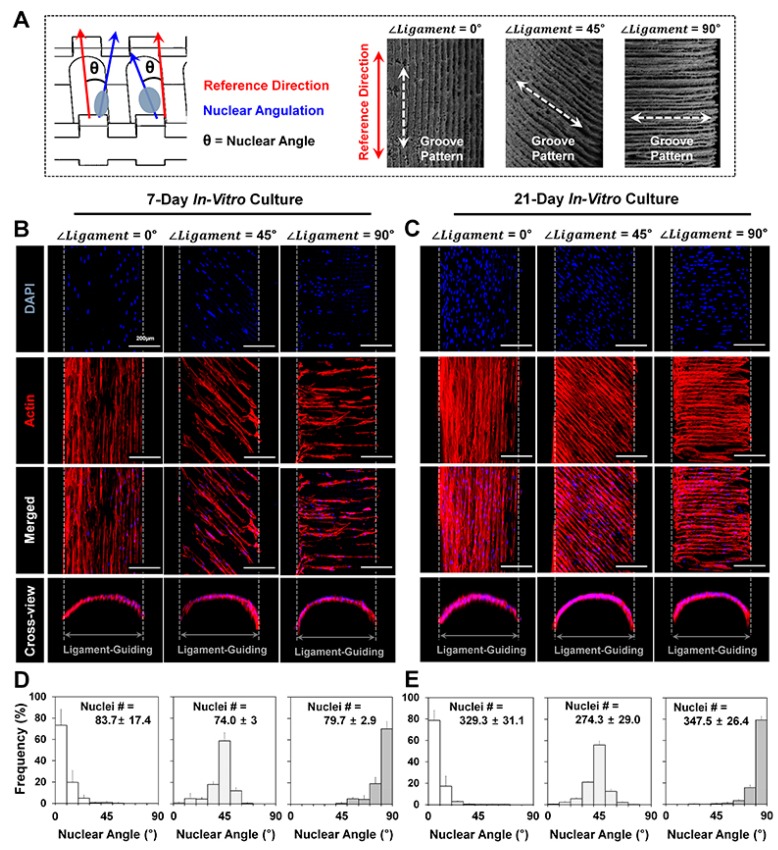
Fluorescence staining method to qualitatively and quantitatively analyze cell and nuclear orientations along microgroove patterns. (**A**) Nuclear angulations were analyzed against the reference direction by 4′,6-diamidino-2-phenylindole (DAPI) staining; (**B**,**C**) the orientations of cellular bundles can be determined by phalloidin-stained actin filaments at 7- and 21-day cultures. Individual microgroove patterns facilitated to angularly organize cells as the in vitro culture period went by ∠*Ligament* = 0°, 45° and 90° with (**D**,**E**) statistical significances of the nuclear angulations. White dash-lines represent the ligament architecture border lines with a 250-μm distance. Nuclei #: the number of analyzed nuclei. Scale bars: 200 μm.

Prior to the investigations of directional cell orientations, the interactions between the microgroove patterns and cell shapes (or nuclear deformations) were required for simple identification of different microgroove patterns, which the 3D printing system can create with a parallel topography to the reference direction (*∠Ligament* = 0°; Figure S2). Slicing thicknesses of 12.70 μm and 25.40 μm were programmed to manufacture wax molds, and identical patterns were found on the spatially-designed PDL architectures after the PCL casting (Figure S2). After hPDL cell culturing with 1.0 × 10^3^ cells per scaffold at 7 days, 12.70-μm microgroove patterns exhibited random organizations of cells with their nuclei; however, the 25.40-μm microgrooves had significantly oriented cell collectivities with typical nuclear angles (frequency of nuclear angle (0–10°) = 73.26 ± 14.85%; Figure S2). In addition, the NAR (nuclear aspect ratio), which is calculated with the long-to-short axis ratio and characterized as one (NAR = 1) for the same long and short axes [23,24], can provide statistical identification of cell shapes and associations with cellular orientations. Based on NSI (nuclear shape index) and NAR, two typical microgroove patterns can markedly guide different morphological characteristics on the same microgroove directionalities. Quantification analyses of the nuclear shapes demonstrated that highly organized cell collectivities on the 25-μm microgrooves had a statistically lower circularity (NSI (12.70 μm) = 0.82 ± 0.039 and NSI (25.40 μm) = 0.65 ± 0.037), which indicates between zero for linear and one for circular shapes (Figure S2). The organization of developed F-actin bundles on spatial structures possessing microgroove patterns was qualitatively observed, and actin cytoskeleton orientations were clearly identified (Figure 5A–C) following the angulated patterns (*∠Ligament* = 0°, 45° and 90°). In addition, the orientations of hPDL cell collectivities were statistically analyzed with the angulations of the anisotropic hPDL nuclei, which strongly corresponded to the microgroove topographies (Figure 5D,E). Approximately 70% or more of the cells were highly aligned on the microgroove patterns on the created topographical-guiding platforms (*∠Ligament* = 0°, 45° and 90°) in the 7- and 21-day cultures (Figure 5A–C and Figure S3). 3D printed microgroove patterns enabled a significant promotion of cell proliferation, as well as directional organization along three different guidable topographies (Figure S3). Based on the results of the increased hPDL populations after the 21-day cultures, the microgroove topographies with rough surfaces by the freeze-casting method facilitated the promotion of cell attachments and proliferation, as well as angulations (Figure 5).

**Figure 6:**
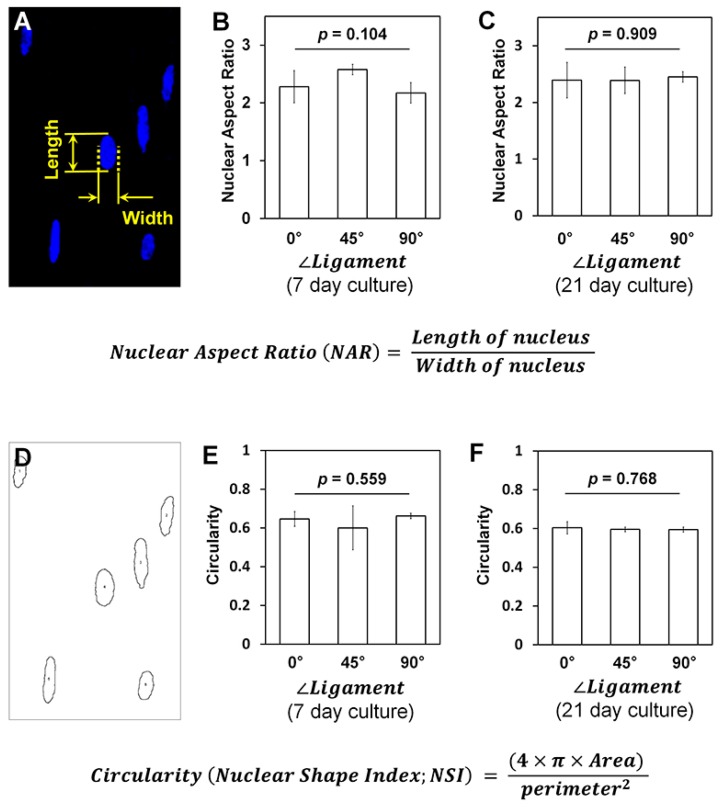
Quantification assessments of nuclear deformations using the nuclear aspect ratio and the nuclear shape index (circularity). (**A**) Nuclei were quantitatively assessed for nuclear deformation using NAR, and (**B**,**C**) three different groups (*∠Ligament* = 0°, 45° and 90°) had no statistically significant differences; (**D**) the NSI can be quantitatively determined for the cell nuclear elongation based on the perimeters of nucleus, and (**E**,**F**) all groups for circularity assessments had statistical similarity (*p*-value > 0.05).

In addition to nuclear angulations, cell shapes consistently correlated with nuclear morphologies, which characterized the microgroove patterns using the analysis methods, NAR and NSI [23,24]. Angulated nuclei were distinctly identified on individual microgroove patterns; however, the NARs and NSIs (or circularities) of ∠*Ligament* of 0°, 45° and 90° had no statistical differences in the 7- and 21-day cultures (*p* > 0.05; Figure 6). As shown in Figure 3 and Figure 4, the nuclei were highly elongated (NAR ~2.34 at 7 days and 2.41 at 21 days) and deformed (NSI ~0.64 at 7 days and 0.59 at 21 days) when the hPDL cells were cultured on angulated microgrooves (Figure 6).

## 3. Discussion

Considering the spatial or dimensional limitations to create scaffolds for large tissue regeneration [25], our investigation could be a possible strategy for manufacturing spatiotemporal architectures with a multitude of microgroove patterns and for creating surface roughness for cell attachments on engineered scaffold surfaces using the freeze-casting method. The appropriate surface properties of 3D structures could improve cell attachment and viability without biochemical modifications given that the surface roughness can regulate cellular activities [26,27]. The roughness on microgroove walls could be acquired by the solvent exchange method with ethanol (T_f_ = −114 °C) and 1,4-dioxane (T_f_ = 11.8 °C) at −20 °C and controlled by using the appropriate concentration of PCL solution (Figure S1). In the quantitative analysis, the 20% and 25% PCL scaffolds had topographical similarities to a typical surface roughness, which Faia-Torres et al. demonstrated to facilitate enhancement of fibroblastic cell attachments and viability without biochemical modifications of the PCL surface [22].

Moreover, the patterned microarchitectures by angulated 3D printing techniques (Figure 3 and Figure 4) can be a key moderator to significantly control cell organizations and tissue morphologies (Figure 5) even though three different types of scaffolds had structural similarities macroscopically (Figure 2E). To evaluate the orientations of scaffold-seeded cell collectivities, significant efforts have contributed to measuring the spatial orientations of a single cell using the cytoskeletal polarity, alignments of actin filaments or deformed nuclear shapes on specifically characterized substrates [23,28,29]. Recently, Versaevel et al. showed an orchestrated correlation between fibroblastic cell orientations and nuclear shapes by the nuclear shape index (NSI; circularity) and the cell shape index on micro-patterned substrates under various mechanistic-regulating microenvironments [24].

Although our model did not apply mechanistic stimulations [24], microgroove patterns can regulate cell orientations by programmed slices and fully-guided nuclear elongations and anisotropic deformations, which are associated with cell orientations [24]. Therefore, the 25-μm microgrooved topography can precisely control cellular orientations as optimal patterns for orientations of cell collectivities. The localized cell adhesion and nuclear elongation/deformation are specifically correlated with F-actin developments in physical-characterized microenvironments, which can also regulate cytoskeleton architectures for cell behaviors [30,31]. In our static condition study, NSI and the nuclear aspect ratio (NAR) were used to measure nuclear orientations corresponding to cell alignments for statistical quantification assessments (Figure 6). Interestingly, the collective cell orientations with high populations were predictably and precisely controlled by the microgroove patterns on the 3D directional ligament scaffolds in the 21-day cultures. At this point, various nano-topographical approaches have demonstrated cell orientations [9,32] or regulations of stem cell responses by anisotropic morphologies or by re-arrangements of cytoskeletal components [33,34]. However, most studies have mainly evaluated individual cell morphologies prior to the increases in cell populations or the formations of strong cell-cell interactions like tissues [32,33].

Based on a simple, but predictable investigation, the technique in this study has strong potential to manufacture spatiotemporal fiber-guiding platforms for 3D fibrous connective tissue formations with specific orientations, which are responsible for functioning restorations in multiple tissue complexes. As examples of the versatilities of 3D printing microgroove patterns, spatial architectures with customized, defect-adapted geometries can be designed for the neogenesis of multiple tissue complexes, which have mineralized structures and fibrous connective tissues in various dimensional interfaces (Figure S4). Based on 3D reconstructed CT-image datasets (Figure S4A), fiber-guiding structures were designed to encompass the tooth-root surfaces for PDL interface regeneration in vivo in the one-wall periodontal osseous defect model (Figure S4B–E). The micro-CT displayed that the customized scaffolds had high adaptability to the created defect geometry (88.30 ± 14.89%; Figure S5), and the SEM showed highly organized and angulated microgroove patterns on the 3D scaffold surfaces (Figure S6).

In general, layer-by-layer artifacts, which are defined as stair stepping errors in the programmed slicing procedure, are commonly removed to increase the surface finish quality and to decrease the critical geometric tolerance for manufacturing accuracy. Recently, 3D printing techniques for biomedical applications have mainly considered cell viability in manufacturing cell-laden spatial constructs with single or multiple cell types [15,35,36]. However, spatiotemporal tissue re-organizations or fibrous re-orientations in engineered microenvironments are the major challenge and are significantly required to restore functionalities in tissue complexes along with cell viabilities [1,5,10]. Our strategy utilized artifactual topographies by the additive manufacturing technique to optimize spatiotemporal cell organizations along engineered directionalities of 3D scaffolds. Microgroove patterns by programmed slicing procedures on the scaffold surface enabled the formation of structural similarities to natural fibrous tissue bundles and fibrous connective tissue orientations with high predictability. In addition, the slice thickness by the additive manufacturing system significantly affected cell organization, and the positional directions to digitally slice designed models were critically influential to create angulated microgroove patterns and to orient cell collectivities with anisotropic nuclear morphologies. Moreover, micron-intervals between the microgrooves that were programmed during the design slices can be significantly correlated to the nuclear deformability regardless of the angulated patterns (Figure S2 and Figure 6). From the perspective of additive manufacturing and 3D printing, spatiotemporal microgroove patterns on 3D printed scaffolds are a promising platform to form hierarchical and functional structures of fibrous connective tissues for tissue engineering and regenerative medicine applications.

## 4. Materials and Methods

### 4.1. Computer Design and Polymeric Fabrication of PDL Scaffolds

For the referential direction of PDL, cylindrical structures (0.5-mm diameter and 3.0-mm length) were designed in a scaffold to comprise multi-layered PDL architectures, opened pores on both sides of a scaffold and two cell-seeding inlets on top (Figure 1) in the CAD (computer-aided design) program, Solidworks 2013 software (Dassault Systems SOLIDWORKS Corp., Waltham, MA, USA). The layer thickness of designed molds was set to 25.40 μm during the digital slicing process, and the designed molds were positioned with three different angles (0°, 45° and 90°) to print wax molds (Figure 2). The additive manufacturing procedure created microgroove patterns with consistent thickness (25.40 μm), which was determined in digital slicing steps (Figure S1). Twenty five percent poly-ε-caprolactone (PCL) solution in 1,4-dioxane was freeze-casted into wax molds, and the solvent was extracted by 99% ethanol and double distilled water at −20 °C for 2 days and 4 °C for 3 days, respectively. Then, wax molds were removed using 35–37 °C cyclohexane for 1 day and, after the PCL scaffolds were cooled down at room temperature, 99% ethanol removed cyclohexane. The PCL scaffolds were stored in 70% ethanol at 4 °C until cell seeding.

### 4.2. Morphological and Topographical Characterizations of Microgroove Patterns on 3D Ligament Architectures

For the qualitative analyses of angulated microgrooves and surface roughness, microgroove patterns on longitudinal architectures in the PCL scaffold were morphologically characterized using the SEM (Figure 2) at 15 kV (S-4700 FE-SEM, Hitachi, Japan). The spatial topographical evaluations were performed for measurements of groove distance (25.40 μm; Figure 3D–F) and angular patterns against the reference direction, which was designed by the CAD for ligament architectures using the topography analysis of the confocal laser scanning microscope (CLSM; Carl Zeiss MicroImaging GmbH, Jena, Germany). The PCL-casted ligament scaffolds were volumetrically and cross-sectionally characterized using micro-CT (SkyScan 1172, Bruker-microCT, Knotich, Belgium), which provide 3D reconstructive and digitally-sectioned images and set to scan with an 11.55 μm^3^ voxel size under a 40 kV source voltage and a 200 μA source current.

### 4.3. In Vitro Cell Culture and Fluorescence Staining for Cell Orientation Analyses

For evaluations of cellular orientations or angulations, human PDL cells (hPDLs) were cultivated in Passages 4–5 with minimum essential medium alpha (α-MEM) including 10% fetal bovine serum (FBS) and antibiotics (100 units/mL penicillin). After seeding 1 × 10^3^ cells into a scaffold containing approximately 30.0 mm^3^, cell-loadable void volume, hPDL-seeded PCL scaffolds were incubated for 7 and 21 days. For nucleus angulation analyses for three different orientations (*∠Ligament* = 0°, 45° and 90°), immunofluorescence was performed with cell nucleus staining with DAPI in blue (4′,6-diamidino-2-phenylindole, Life Technologies (Thermo Fischer Scientific, Waltham, MA, USA) and F-actin staining with phalloidin in red (Alexa Fluoro^®^ 546 Phalloidin, Life Technologies, Carlsbad, CA, USA) after fixing cultured cells at different time points. Using ImageJ software (National Institutes of Health (NIH), Bethesda, MD, USA), angulations of DAPI-stained cell nucleus were measured against the reference direction, which was defined by the designed architecture (Figure 5A–C).

### 4.4. Nuclear Shape and Deformation Analyses

To quantitatively analyze nuclear deformations by microgroove patterns and microgroove angulations, projected DAPI-stained images were utilized to calculate nuclear aspect ratio (NAR), which was calculated with measured long and short axes of each nucleus and circularity (nuclear shape index (NSI)), which was analyzed with perimeters and area by the ImageJ software.

### 4.5. 3D Customized Scaffold Developments with Geometric Adaptation to the 1-Wall Periodontal Defect

After scanning the dissected cadaveric mandible by micro-CT (SkyScan 1172, Bruker microCT, Kontich, Belgium), we digitally created the 1-wall periodontal defect. The Solidworks 2013 software was utilized to design fiber-guiding scaffolds with PDL and bone architectures. The Magics 19 software (Materialise Inc., Leuven, Belgium) was utilized to generate defect-fit geometries of scaffolds by booleaning two-image data, which were the 3D reconstructed 1-wall defect and a computer-designed scaffold. 3D printed wax molds having designed scaffold architectures had the freeze-casting procedure using 25% PCL in 1,4-dioxane. Ninety nine percent ethanol and double-distilled water-extracted frozen 1,4-dioxane solvent at −20 °C for 2 days and 4 °C for 2 days, respectively, were used. After removing wax molds by cyclohexane at 35–37 °C, 99% ethanol was used to remove residual cyclohexane in PCL scaffolds at room temperature, and scaffolds were stored in 70% ethanol at 4 °C. For SEM scanning, the solvent was changed to double-distilled water, and the PCL scaffolds were freeze-dried.

### 4.6. Statistical Analysis 

All data were analyzed using the mean ± standard deviation (SD). For comparisons of the nuclear aspect ratio (NAR) and circularity (nuclear shape index (NSI) with three different groups (*∠Ligament* = 0°, 45° and 90°), the one-way analysis of variance (one-way ANOVA) test with Bonferroni correction was utilized with the α-value set at the 0.05 level of significance.

## 5. Conclusions

3D printing technologies have been rapidly developed to create various cell-viable hierarchical architectures for tissue engineering, but it is still challenging to spatiotemporally control the orientations of ligamentous bundles for physiological functioning restorations in musculoskeletal complexes. We demonstrate that the different angulated microgroove patterns on 3D printed scaffolds can control the orientation of ligamentous cell bundles with high manufacturing reproducibility. This simple strategy provides the topographical platform to precisely form functional architectures for 3D organizations of fibrous connective tissues. The ligament-guiding architectures can integrate designed bone compartments to create ligament-bone constructs, and the multi-compartmentalized constructs can lead to tissue-functioning restoration with multiple tissue neogenesis in in vivo biomedical applications.

## Supplementary Materials

Supplementary materials can be found at www.mdpi.com/1422-0067/18/9/1927/s1.

## Abbreviations

hPDL	Human periodontal ligament
CAD	Computer-aided design
PCL	Poly-ε-caprolactone
SEM	Scanning electron microscopy
Micro-CT	Micro-computed tomography
CLSM	Confocal laser scanning microscopy
DAPI	4′,6-Diamidino-2-phenylindole
Ra	Arithmetic roughness average
T_f_	Freezing temperature
NSI	Nuclear shape index
NAR	Nuclear aspect ratio
