# Supplementary material for: 3D Printed, Microgroove Pattern-Driven Generation of Oriented Ligamentous Architectures
Source: Int J Mol Sci. 2017 Sep 8;18(9):1927. doi: 10.3390/ijms18091927 (PMC5618576; doi:10.3390/ijms18091927)
Supplement: Supplementary file 1 [file ijms-18-01927-s001.pdf]

Supplementary Materials

3D Printed, Microgroove Pattern-Driven Generation of Oriented Ligamentous Architectures

Chan Ho Park*, Kyoung-Hwa Kim, Yong-Moo Lee, William V. Giannobile, and Yang-Jo Seol*

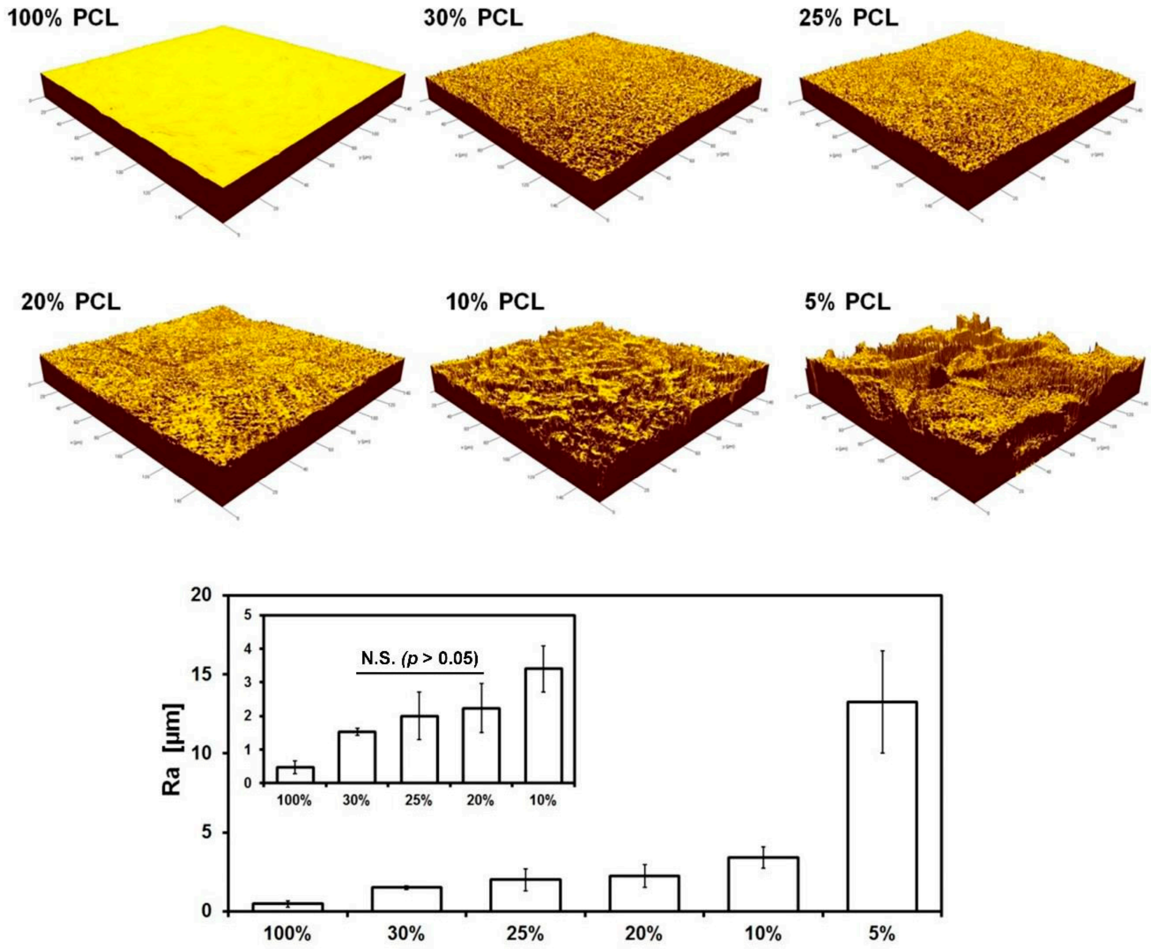

Figure S1. Surface topography analyses of 2D PCL platforms by the freeze-casting method with various concentrations of PCL solutions. 2D polymer surface analysis was utilized as the alternative method because the spatiotemporal architectures or 3D platforms with various micro-roughness had critical limitations to characterize the microarchitecture. Various concentrations of PCL solution within 1,4-dioxane solvent were attempted to generate different roughnesses using the freeze-casting method in wax molds and can identify significantly different roughness values (Ra), which can be represented for surface topographies. Except for the 5% PCL surface that had significantly high roughness, 30%, 25% and 20% PCL

surfaces had statistically similar roughness and topographies, such as 1.529 ± 0.112, 2.003 ± 0.700 and 2.237 ± 0.727, respectively (N.S., no significant difference).

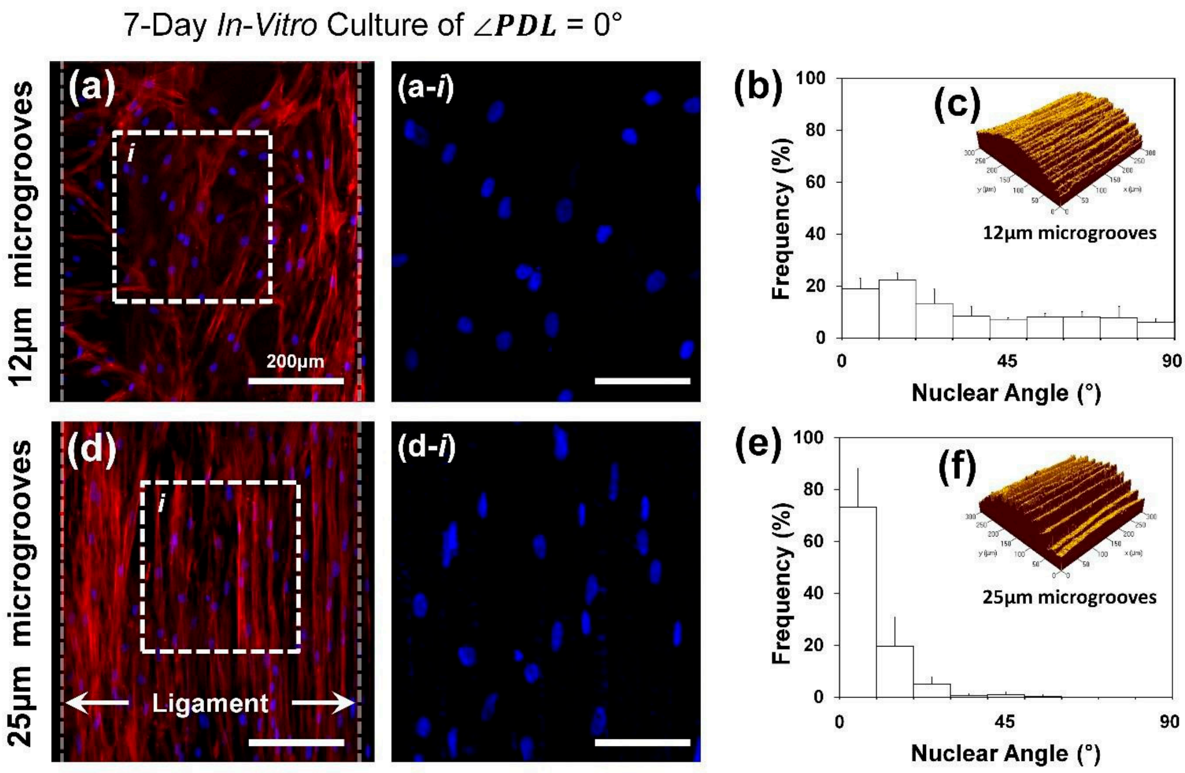

Microgroove ($\angle PDL$ = 0°)	Circularity (Nuclear Shape Index; NSI)	Nuclear Aspect Ratio
**12μm microgrooves (n=3)**	**0.82 ± 0.039 *** **	**1.56 ± 0.11 ** **
**25μm microgrooves (n=3)**	**0.65 ± 0.037 *** **	**2.28 ± 0.28 ** **

Figure S2. Statistical correlations between the microgroove patterns and cell orientations. Using different slicing thicknesses with 12 μm and 25 μm, the additive manufacturing system created microgroove patterns on ligament-guiding structures as the topographical images showed. After cell seeding and culturing for seven days, each microgroove pattern distinctly identified the differences of cell orientations and morphologies (merged fluorescence images), as well as nuclear deformations (DAPI-fluorescence images and Table).

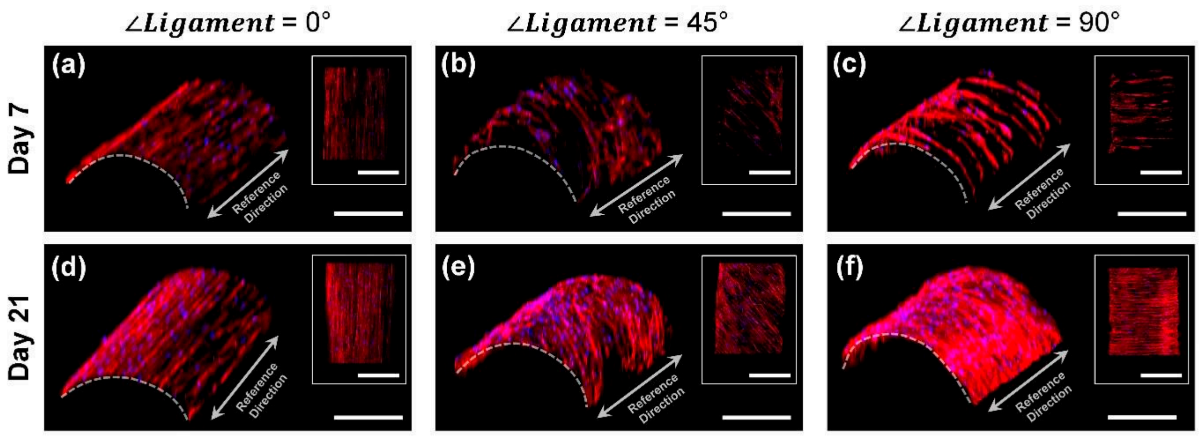

Figure S3. 3D reconstructed images of immunofluorescence-stained fibrous cells on microgroove patterned scaffolds. The confocal z-stack images and 2D projection images on the right side of the 3D images show that immunofluorescence-stained cells were aligned with specific orientations. DAPI-stained cell nuclei and phalloidin-stained actin filaments were highly organized with the parallel, oblique and perpendicular orientations by microgroove patterns on ligament scaffolds against the reference direction, which was defined as the CAD-based ligament architecture. Scale bar: 300 μm.

Supporting Methods

3D customized scaffold developments with geometric adaptation to the 1-wall periodontal defect:

After scanning the dissected cadaveric mandible by micro-CT (SkyScan), we digitally created the 1-wall periodontal defect. The Solidworks 2013 software was utilized to design fiber-guiding scaffolds with PDL and bone architectures. The Magics 19 software (Materialise Inc. Leuven, Belgium) was utilized to generate defect-fit geometries of scaffolds by booleaning two image data, which were the 3D reconstructed 1-wall defect and a computer-designed scaffold. 3D printed wax molds having designed scaffold architectures had the freeze-casting procedure using 25% PCL in 1,4-dioxane. Ninety-nine percent ethanol and double-distilled water-extracted frozen 1,4- dioxane solvent at −20 °C for 2 days and 4 °C for 2 days, respectively, were used. After removing wax molds by cyclohexane at 35–37 °C, 99% ethanol was used to remove residual cyclohexane in PCL scaffolds at room temperature, and scaffolds were stored in 70% ethanol at 4°C. For SEM scanning, the solvent was changed to double-distilled water, and PCL scaffolds were freeze-dried.

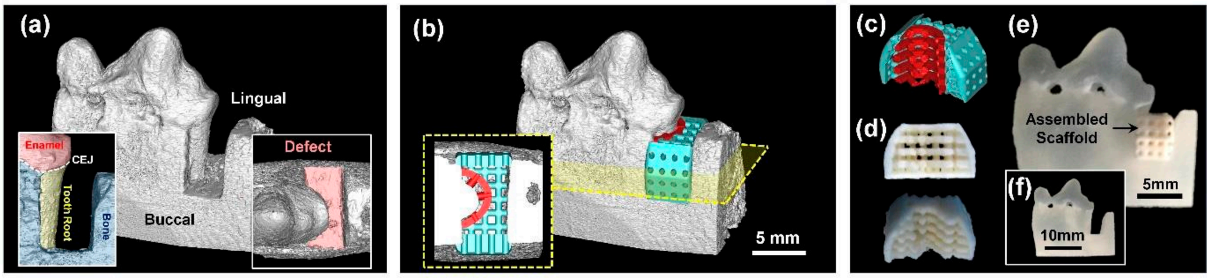

Figure S4. The periodontal one-wall defect model by the reverse-engineering technology to manufacture 3D scaffolds. (**a**) After micro-CT scanning, the periodontal one-wall defect canine model, (**b**) the customized 3D scaffold was designed with compartmentalized architectures for periodontal ligament (PDL; red color) and alveolar bone (blue color). (**c**) The 3D wax printer manufactured molds based on the computer-designed architecture and (**d**) the biopolymeric PCL material was cast for spatial PCL platforms for periodontal complex neogenesis. (**e**–**f**) Fabricated PCL scaffolds were placed into the one-wall defect of the 3D printed prototyped model.

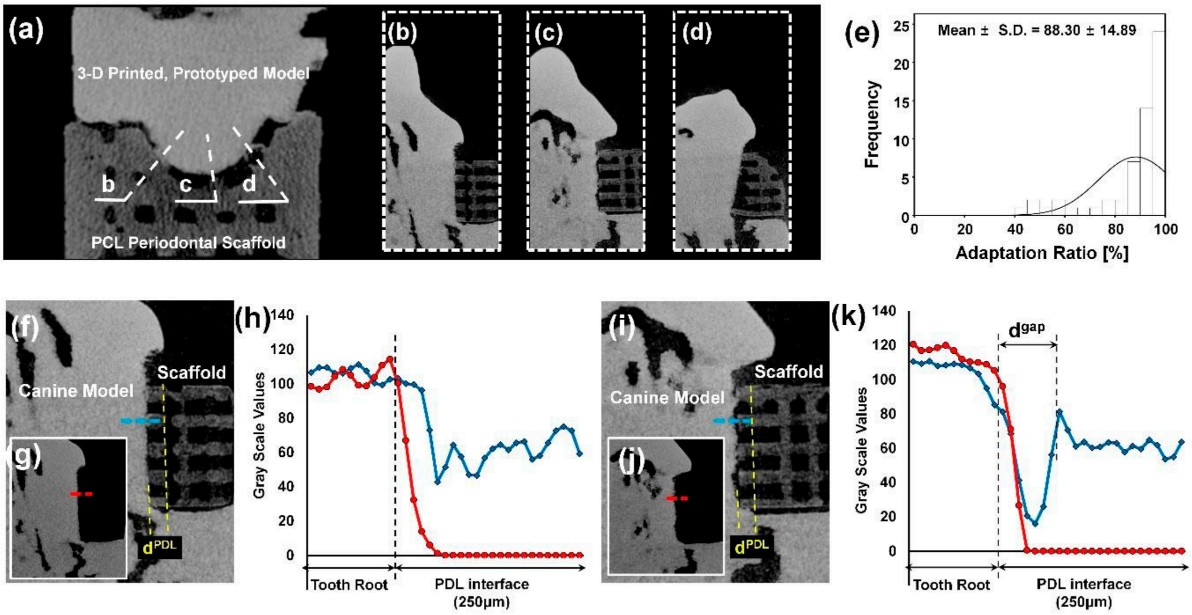

Figure S5. (**a**) PDL structures had perpendicular directionalities with geometrical adaptations to the tooth root surface by micro-CT with 2D coronal and (**b**–**d**) sagittal section images. (**e**) For the adaptation analysis, the gap distance between the individual PDL architecture and root surface was measured, and a high adaptation ratio (88.30% ± 14.89) was calculated. (**f**–**g**) The architectural analysis using the geometric adaptation ratio. (**f**–**h**) Using the reconstructed micro-CT image dataset, which was obtained after scanning 3D printed canine models with/without scaffolds, the geometric adaptability was analyzed, and the adaptation ratio was calculated using the grayscale-based histograms from the part of the tooth root. (**i**–**k**) The adaptation ratio was calculated with the gap distance (dgap), which can be determined as the low adaptable architecture to the tooth root defect. (**k**) Using the histogram, the adaptation ratio was calculated with the measured dgap and the length of PDL (200 < dPDL (μm) < 300), which can be individually identified during the measurements of architectures, and the histogram was from 70.0% adaptable scaffold image. (**f**–**k**) Yellow dashed lines represent the borderline of the PDL interface; red-dash lines the linear measurement without the scaffold; and blue dashed lines for the measurement from the scaffold-placed model image. (**h**, **k**) The blue and red lines in the histograms are plotted from the blue dashed and red dashed lines in the micro-CT images.

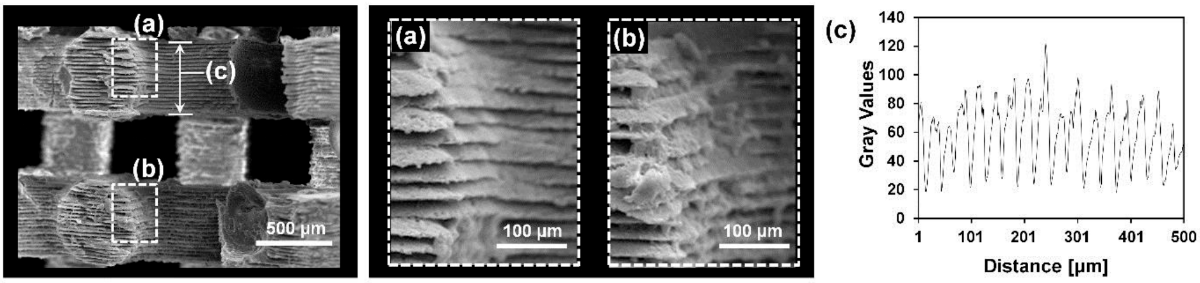

Figure S6. The qualitative analysis using SEM images demonstrated the parallel microgroove patterns to PDL architectures with low magnification and (**a**–**c**) high magnification, which had 25.40-μm intervals on the structure surfaces. The microgroove patterned architectures had qualitative similarity to ligament scaffolds and can be the key strategy to topographically guide ligament fibers. CEJ: cemento-enamel junction; mean ± SD (standard deviation); and the total number of analyzed ligament (PDL) structures (n) = 57.
